# Vapor–Liquid Equilibrium Study of LiBr + H_2_O and LiBr + CaCl_2_ + H_2_O Systems

**DOI:** 10.3389/fchem.2019.00890

**Published:** 2020-01-23

**Authors:** Huinan Wang, Hongfei Chen, Wanhao Chen, Haoran Sun, Xianzhen Xu

**Affiliations:** Laboratory of Fiber Materials and Modern Textile, Shandong Sino-Japanese Center for Collaborative Research of Carbon Nanomaterials, Collaborative Innovation Center for Marine Biomass Fiber Materials and Textiles, College of Chemistry and Chemical Engineering, Qingdao University, Qingdao, China

**Keywords:** electrolyte solution, vapor–liquid equilibrium (VLE), measurement, modeling, thermodynamics

## Abstract

Vapor–liquid equilibrium (VLE) data and modeling for LiBr + H_2_O and LiBr + CaCl_2_ + H_2_O are reported in this paper. This work focuses on the experimental determination of the boiling point of LiBr + H_2_O and LiBr + CaCl_2_ + H_2_O solutions with vapor pressures between 6 and 101.3 kPa and the total molality of salt ranging from 0 to 21.05 mol•kg^−1^. The procedures were carried out in a computer-controlled glass apparatus. The relationship between the boiling point and saturated vapor pressure is obtained, and Xu's model is used to correlate and predict the VLE. By correlation of the data (literature and experimental) for LiBr + H_2_O and LiBr + CaCl_2_ + H_2_O, the parameters are obtained. We compared the results with the ElecNRTL model and Pitzer model. The parameters for the LiBr + H_2_O, CaCl_2_ + H_2_O, and LiBr + CaCl_2_ + H_2_O systems can be successfully used to calculate and predict the VLE data.

## Introduction

The vapor–liquid equilibrium (VLE) of electrolyte sol utions is widely used in industries, natural processes, chemistry, and chemical engineering. LiCl, LiBr, and CaCl_2_ aqueous solutions have extensive applications in the field of refrigeration, cooling, and heat transforming systems based on absorption cycles (Lan et al., 2017; Li et al., 2017). Simultaneously, the thermodynamic properties of the solutions play a key role in the absorption cycles.

Due to the strong demand for absorption and separation process design, an increasing number of researchers have studied the VLE of electrolyte systems. Massive quantities of phase equilibrium data have been reported in recent years. Some solubility isotherms of the LiCl + CaCl_2_ + H_2_O system have been measured (Filippov and Mikhelson, 1977; Zeng et al., 2008), and VLE data of LiCl + H_2_O, CaCl_2_ + H_2_O, and LiCl + CaCl_2_ + H_2_O systems have been obtained (Xu et al., 2014, 2019a). Lan et al. (2017) and N'Tsoukpoe et al. (2013) experimentally determined the saturated vapor pressure of LiBr aqueous solution with mass fractions ranging from 43.14 to 65.26 wt.% at high temperature. Chua et al. (2000) presented a thermodynamically consistent set of specific enthalpy, entropy, and heat capacity fields for a LiBr + H_2_O solution. However, the phase equilibrium data of the systems containing LiBr with a wide range of pressures and temperatures are still rare.

The experimental data and thermodynamic models are equally important (Xu et al., 2019b). Significant improvements have been made in calculating thermodynamic properties using theoretical models; most models are based on the Wilson model (Wilson, 1964), NRTL model (Renon and Prausnitz, 1968), and UNIQUAC model (Abrams and Prausnitz, 1975). For electrolyte solutions, the Pitzer model (Pitzer, 1973), ElecNRTL model (Chen et al., 1982; Chen and Evans, 1986), Lu–Maurer model (Lu and Maurer, 1993; Lu et al., 1996), extended UNIQUAC model (Thomsen et al., 1998), and Xu model (Xu et al., 2016, 2019c) have been widely utilized. The thermodynamic properties of the binary systems (CaCl_2_ + H_2_O and LiCl + H_2_O) were simulated by the Pitzer–Simonson–Clegg (PSC) model in Li's work (Li et al., 2015, 2016). Patek and Klomfar (2006) developed an effective formulation of the thermodynamic properties of LiBr–H_2_O solutions from 273 to 500 K. Despite the aforementioned research works, thermodynamic property calculations for some electrolyte systems still face great challenges, and more accurate models over a wide range of pressures, temperatures, and concentrations are needed.

Due to the lack of VLE data for systems containing LiBr at a wide range of pressures and temperatures, in this work, VLE data of LiBr + H_2_O and LiBr + CaCl_2_ + H_2_O systems are experimentally measured at concentrations ranging from 0 to 21.05 mol•kg^−1^ and pressures ranging from 6 to 101.3 kPa. In addition, the obtained data are used to parameterize Xu's model (Xu et al., 2016). We expand the scope of the model, such as concentration, pressure, and temperature. Correlation and prediction of the VLE of LiBr + H_2_O and LiBr + CaCl_2_ + H_2_O were successfully developed.

## Experimental Section

### Materials

Anhydrous LiBr (purity ≥ 99.5%) and anhydrous CaCl_2_ (purity ≥ 99.99%) were purchased from Adamas-Beta. Distilled water (18.2 Ω cm) was used for the preparation of solutions.

### Apparatus and Procedures

**Figure 1:**
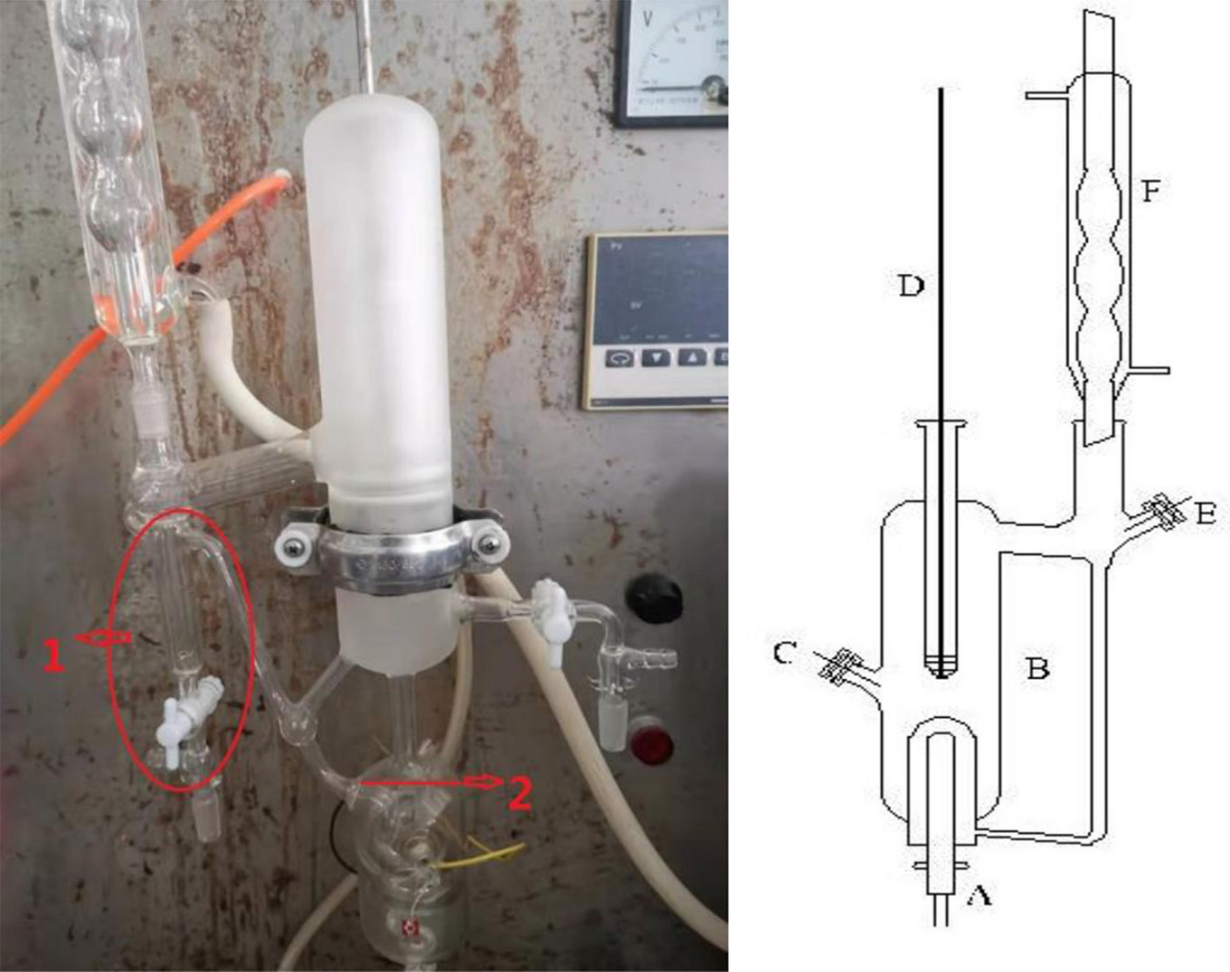
A dual circulation glass ebulliometer.

A dual circulation glass ebulliometer (40 ml) was used in the VLE measurements, as shown in Figure 1. The main experimental instruments are listed in Table 1, including a vacuum pump in the ebulliometer, a pressure controller, a heating mantle, and a temperature controller.

**Table 1:** The main experimental instruments.

**Instrument**	**Model**	**Manufacturer**	**Uncertainty**
Dual circulation glass ebulliometer	40 cm^3^	Tianjin Wuqing Beiyang Chemical Factory	
Pressure controller	Ruska Series 7000 controller	Ruska Instrument Corp., Houston, USA	±0.01 kPa
Temperature controller	Model SRS13A	SHIMADEN, Japan	±0.05 K
Electronic balances	SECURA225D-1CEU balances	Sartorius Lab Instruments GmbH & Co. KG 37070 Gorttingen, Germany	±0.0001 g

**Figure 2:**
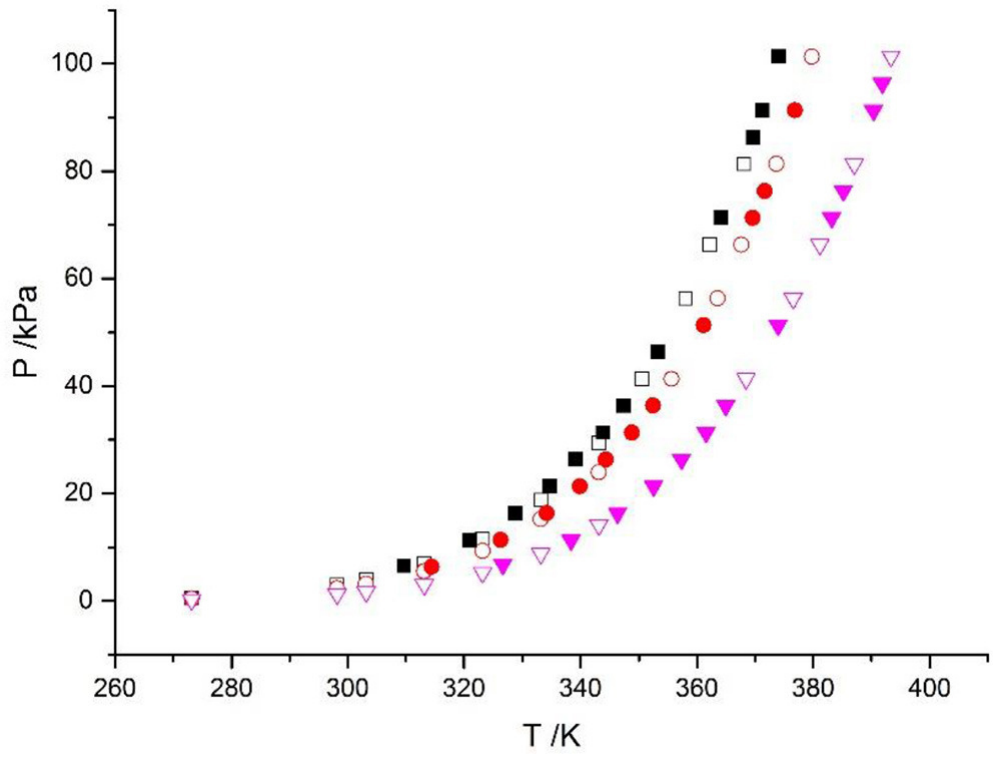
Vapor-liquid equilibrium in the CaCl_2_ + H_2_O system. Empty symbols (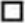, *m* = 1 mol/kg; 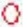, *m* = 3 mol/kg; 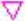, *m* = 6 mol/kg): literature data (Xu et al., 2014, 2016; Lan et al., 2017); full symbols (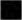, *m* = 1 mol/kg; 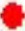, *m* = 3 mol/kg; 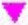, *m* = 6 mol/kg): experimental data (Xu et al., 2014).

The reliability of the experiment has been verified in the literature (Xu et al., 2014, 2019a) (i.e., CaCl_2_ + H_2_O and NaCl + KCl + H_2_O), as shown in Figure 2. The experimental data for the LiBr + H_2_O and LiBr + CaCl_2_ + H_2_O systems at different molalities are listed in Tables 2–6. Each VLE experimental data in this work are averages taken after three experiments. For the systems containing LiBr, the solubility of the salt is relatively high, with a value of 21.05 mol•kg^−1^ at 298.15 K. The absorption is relatively strong at higher salt concentrations. The pressure (6–101.3 kPa) is an important factor for the design of absorption and separation processes.

**Table 2:** Experimental VLE data for temperature *T*, pressure *P*, and molality *m—*LiBr for the LiBr + H_2_O system.

***m*** **=** **21.05 mol**•**kg**^**-1**^		***m*** **=** **16.5 mol**•**kg**^**-1**^		***m*** **=** **11 mol**•**kg**^**-1**^		***m*** **=** **9.01 mol**•**kg**^**-1**^	
***T* (K)**	***P* (kPa)**	***T* (K)**	***P* (kPa)**	***T* (K)**	***P* (kPa)**	***T* (K)**	***P* (kPa)**
319.65	6.29	318.35	6.405	333.15	6.3	324.75	5.94
332.25	11.875	329.45	11.33	345.05	10.955	338.35	11.085
340.85	16.38	337.35	16.265	354.55	16.61	347.95	16.405
347.15	21.3	343.35	21.365	360.95	21.185	354.25	21.495
352.05	26.205	347.95	26.21	366.35	26.225	359.45	26.05
356.45	31.075	352.35	31.295	371.25	31.235	363.85	30.94
360.45	36.075	356.05	36.2	375.35	36.23	368.25	36.08
363.85	41.23	359.45	41.34	378.95	41.27	371.95	41.07
366.85	45.98	362.25	46.14	382.05	46.38	375.05	46.155
369.55	50.96	364.85	51.26	384.95	51.445	378.05	51.24
372.25	56.165	367.55	56.34	387.65	56.3	380.85	56.25
374.55	60.865	369.95	61.305	390.05	61.51	383.45	61.33
376.75	66.715	371.95	66.18	392.45	66.575	385.55	66.22
378.85	70.91	374.05	70.78	394.65	71.02	387.55	71.035
380.85	76.2	376.15	75.75	396.55	75.885	389.75	76.63
382.65	80.975	377.85	81.11	398.05	81.205	391.45	81.26
384.45	86.145	379.85	86.355	399.95	86.255	393.25	86.31
386.05	91.315	381.45	91.26	401.95	91.27	395.15	91.29
387.65	96.12	383.05	96.165	403.25	96.265	396.75	96.26
389.15	101.255	384.45	101.235	404.55	101.245	398.25	101.245

*Standard uncertainties u are u(P) = 0.1 kPa, u(T) = 0.05 K, u(m) = 0.0001 g, respectively*.

**Table 3:** Experimental VLE data for temperature *T*, pressure *P*, and molality *m—*LiBr for the LiBr + H_2_O system.

***m*** **=** **7 mol**•**kg**^**-1**^		***m*** **=** **4.8 mol**•**kg**^**-1**^		***m*** **=** **4 mol**•**kg**^**-1**^		***m*** **=** **1.5 mol**•**kg**^**-1**^	
***T* (K)**	***P* (kPa)**	***T* (K)**	***P* (kPa)**	***T* (K)**	***P* (kPa)**	***T* (K)**	***P* (kPa)**
319.65	6.29	318.35	6.405	316.95	6.51	311.65	6.59
332.25	11.875	329.45	11.33	326.95	11.29	322.45	11.59
340.85	16.38	337.35	16.265	334.35	16.105	329.35	16.245
347.15	21.3	343.35	21.365	340.35	21.29	335.25	21.24
352.05	26.205	347.95	26.21	344.85	26.4	339.85	26.31
356.45	31.075	352.35	31.295	348.95	31.685	343.75	31.175
360.45	36.075	356.05	36.2	352.65	36.345	347.45	36.31
363.85	41.23	359.45	41.34	355.55	41.405	350.95	41.495
366.85	45.98	362.25	46.14	358.45	46.485	353.45	46.22
369.55	50.96	364.85	51.26	361.15	50.965	356.05	51.24
372.25	56.165	367.55	56.34	363.55	56.36	358.45	56.425
374.55	60.865	369.95	61.305	365.85	61.45	360.65	61.25
376.75	66.715	371.95	66.18	368.15	66.235	362.75	66.155
378.85	70.91	374.05	70.78	370.35	71.355	364.65	71.31
380.85	76.2	376.15	75.75	372.15	76.435	366.45	76.295
382.65	80.975	377.85	81.11	373.95	81.22	368.35	81.345
384.45	86.145	379.85	86.355	375.65	86.105	370.05	86.325
386.05	91.315	381.45	91.26	377.65	91.5	371.65	91.185
387.65	96.12	383.05	96.165	378.55	96.155	373.25	96.375
389.15	101.255	384.45	101.235	380.25	101.235	374.65	101.325

*Standard uncertainties u are u(P) = 0.1 kPa, u(T) = 0.05 K, u(m) = 0.0001 g, respectively*.

**Table 4:** Experimental VLE data for temperature *T*, pressure *P*, and molality *m* (*ma—*LiBr, *mb—*CaCl_2_) for the LiBr + CaCl_2_ + H_2_O system.

***ma*** **=** **21.05 mol**•**kg**^**-1**^ ***mb*** **=** **0 mol**•**kg**^**−1**^		***ma*** **=** **1.5 mol**•**kg**^**-1**^ ***mb*** **=** **8.5 mol**•**kg**^**-1**^		***ma*** **=** **3.08 mol**•**kg**^**−1**^ ***mb*** **=** **8.1 mol**•**kg**^**-1**^		***ma*** **=** **4.12 mol**•**kg**^**-1**^ ***mb*** **=** **7.1 mol**•**kg**^**-1**^	
***T* (K)**	***P* (kPa)**	***T* (K)**	***P* (kPa)**	***T* (K)**	***P* (kPa)**	***T* (K)**	***P* (kPa)**
362.25	6.425	337.55	6.395	338.65	6.475	337.95	5.98
375.95	11.295	349.65	11.165	351.35	11.445	352.25	11.425
384.95	16.17	357.95	16.18	359.95	16.27	360.95	16.415
391.95	21.31	364.15	21.305	366.35	20.955	366.75	21.08
398.15	26.78	368.85	26.14	371.55	26.195	371.95	26.465
401.95	31.48	373.25	31.28	376.35	31.315	376.35	31.47
404.95	35.5	376.45	36.145	380.05	36.31	380.45	36.315
408.45	40.55	379.75	41.1	382.25	41.22	383.55	40.975
410.65	45	382.35	46.32	384.45	46.345	386.65	46.43
413.35	51.055	384.55	51.22	386.95	51.235	389.25	51.26
416.35	56.455	386.85	56.385	389.55	56.155	392.05	55.95
418.65	60.87	389.45	61.25	391.95	61.27	394.15	61.16
420.55	66.18	392.35	66.84	394.35	66.245	396.25	65.83
422.45	70.975	394.15	71.165	395.55	71.865	398.85	71.2
424.15	76.595	395.75	76.385	397.35	75.785	401.25	76.33
425.35	81.26	397.75	81.47	398.45	81.33	402.75	81.125
426.45	86.265	399.25	86.19	399.25	86.355	404.35	86.33
427.65	91.445	400.85	91.265	400.25	91.31	405.65	91.33
428.75	96.74	401.85	96.27	402.35	96.32	407.25	96.34
429.65	101.225	403.45	101.235	402.75	101.205	408.35	101.215

*Standard uncertainties u are u(P) = 0.1 kPa, u(T) = 0.05 K, u(m) = 0.0001 g, respectively*.

**Table 5:** Experimental VLE data for temperature *T*, pressure *P*, and molality *m* (*ma—*LiBr, *mb—*CaCl_2_) for the LiBr + CaCl_2_ + H_2_O system.

***ma*** **=** **5.5 mol**•**kg**^**-1**^ ***mb*** **=** **6.1 mol**•**kg**^**-1**^		***ma*** **=** **7.1 mol**•**kg**^**-1**^ ***mb*** **=** **4.7 mol**•**kg**^**-1**^		***ma*** **=** **8.95 mol**•**kg**^**−1**^ ***mb*** **=** **4.08 mol**•**kg**^**-1**^		***ma*** **=** **11 mol**•**kg**^**-1**^ ***mb*** **=** **3.3 mol**•**kg**^**-1**^	
***T* (K)**	***P* (kPa)**	***T* (K)**	***P* (kPa)**	***T* (K)**	***P* (kPa)**	***T* (K)**	***P* (kPa)**
338.35	6.33	336.85	6.05	342.45	6.2	342.65	6.145
349.85	10.935	351.95	11.47	355.85	11.095	357.45	11.29
359.25	16.14	360.15	16.145	365.25	16.465	366.25	16.17
366.05	21.285	368.85	21.365	372.05	21.31	373.05	21.385
371.85	26.21	373.65	25.91	377.45	26.205	379.25	26.18
376.25	31.47	378.95	31.255	382.05	30.935	384.25	31.135
380.05	36.41	382.85	35.87	386.05	36.12	388.05	38.31
383.45	41.37	386.95	41.225	389.95	41.255	391.65	40.875
386.45	46.425	390.35	46.09	392.55	46.245	395.15	46.28
388.95	51.01	393.25	51.085	395.35	50.98	398.05	51.445
391.85	56.31	395.85	56.265	398.15	56.05	400.75	56.175
394.65	62.045	398.65	61.32	400.55	61.39	403.45	61.26
396.35	66.41	401.05	65.955	402.75	66.225	405.25	65.89
398.15	71.4	402.55	71.155	405.15	71.005	407.15	71.31
399.75	76.48	404.75	76.185	407.15	76.435	408.95	74.16
400.95	81.27	406.85	81.28	408.45	81.255	410.75	81.31
402.65	86.205	408.35	86.185	409.95	86.395	412.95	86.175
403.95	91.285	410.15	91.385	411.45	91.36	415.05	91.265
404.55	96.29	411.45	96.34	412.85	96.35	416.45	96.405
405.65	101.315	412.75	101.185	415.65	101.205	416.85	101.215

*Standard uncertainties u are u(P) = 0.1 kPa, u(T) = 0.05 K, u(m) = 0.0001 g, respectively*.

**Table 6:** Experimental VLE data for temperature *T*, pressure *P*, and molality *m* (*ma—*LiBr, *mb—*CaCl_2_) for the LiBr + CaCl_2_ + H_2_O system.

***ma*** **=** **16.6 mol**•**kg**^**-1**^ ***mb*** **=** **1.5 mol**•**kg**^**-1**^		***ma*** **=** **0 mol**•**kg**^**-1**^ ***mb*** **=** **8.91 mol**•**kg**^**-1**^	
***T* (K)**	***P* (kPa)**	***T* (K)**	***P* (kPa)**
350.85	6.35	336.55	6.395
366.45	11.51	348.55	11.375
377.15	17.65	356.85	16.2
381.15	19.75	363.35	21.405
385.45	23.835	368.25	26.235
390.75	28.625	372.25	31.565
394.85	33.67	376.15	36.325
400.15	38.815	379.65	41.295
403.55	44.15	382.85	46.26
406.85	48.96	385.45	51.2
409.55	54.155	387.75	56.31
412.05	58.805	390.75	61.41
413.95	63.71	392.95	66.27
415.85	68.79	394.85	71.475
417.95	74.95	396.85	76.49
419.55	80.885	398.45	81.21
421.05	86.415	400.25	86.175
422.15	90.845	401.75	91.085
423.65	96.35	403.15	96.275
425	101.225	404.45	101.245

*Standard uncertainties u are u(P) = 0.1 kPa, u(T) = 0.05 K, u(m) = 0.0001 g, respectively*.

The experimental procedures are as follows: (1) During the experiments, the sample was placed into the glass ebulliometer. When we were ready to add the sample into the ebulliometer, we filled the sample solution in the part marked 1 in Figure 1. Because of the problem of water condensation, if the part was not filled with the sample solution, the experimental results would have a large error. The sample should be added to the height of mark 2 shown in Figure 1. (2) The ebulliometer was heated by the heating mantle and was controlled by the voltage controller. (3) The operation pressure was controlled by the vacuum pump, the pressure sensor, and the control valve. (4) After the sample was added, we turned on the heater and controlled the heating voltage. Then, we stably controlled the pressure in the ebulliometer through the pressure controller. (5) The vapor H_2_O was condensed in a spherical condenser (length 40 cm) and then returned to the mixing chamber for recirculation. The time was 0.5–1 h in the first equilibrium, and the following equilibrium time was 10–20 min. The judging standard of the VLE is an important factor. The condensate reflux of the ebulliometer was controlled at two to three drops per second and was stably refluxed for ~15 min to establish an equilibrium state. (6) After the VLE was reached, we recorded the temperature and pressure.

## Model Description

### Xu Model

In the Xu model (Xu et al., 2016) for mixed electrolyte solution systems, the equation was based on the NRTL model:

(1) $$\begin{array}{l} \frac{n_{t}G_{NRTL}^{e}}{RT}=m_{x}m_{w}\left(\frac{\tau_{w,x}G_{w,x}}{m_{x}+m_{w}G_{w,x}}+\frac{\tau_{x,w}G_{x,w}}{m_{w}+m_{x}G_{x,w}}\right) \end{array}$$

(2) $$\begin{array}{l} G_{w,x}=\exp\left(-\alpha \tau_{w,x}\right) \end{array}$$

(3) $$\begin{array}{l} G_{x,w}=\exp\left(-\alpha \tau_{x,w}\right) \end{array}$$

(4) $$\begin{array}{l} m_{w}=\frac{1000}{M\text{s}}-\overset{n}{\underset{i=1}{\sum }}\left(h_{i}m_{i}\right) \end{array}$$

(5) $$\begin{array}{l} \tau_{w,x}=\overset{n}{\underset{i=1}{\sum }}\left(\tau_{w,i}m_{i}\right)/\overset{n}{\underset{i=1}{\sum }}\left(m_{i}\right) \end{array}$$

(6) $$\begin{array}{l} \tau_{x,w}=\overset{n}{\underset{i=1}{\sum }}\left(\tau_{i,w}m_{i}\right)/\overset{n}{\underset{i=1}{\sum }}\left(m_{i}\right) \end{array}$$

(7) $$\begin{array}{l} \tau_{w,i}=\tau_{w,i}^{\left(0\right)}+\tau_{w,i}^{\left(1\right)}/T \end{array}$$

(8) $$\begin{array}{l} \tau_{i,w}=\tau_{i,w}^{\left(0\right)}+\tau_{i,w}^{\left(1\right)}/T \end{array}$$

The final equation can be written as:

(9) $$\begin{array}{l} \ln\;a_{w}\text{=}\left(\frac{\overset{n}{\underset{i=1}{\sum }}\left(\tau_{w,i}m_{i}\right)G_{w,x}}{\overset{n}{\underset{i=1}{\sum }}\left(m_{i}\right)+m_{w}G_{w,x}}+\frac{\overset{n}{\underset{i=1}{\sum }}\left(\tau_{i,w}m_{i}\right)G_{x,w}}{m_{w}+\overset{n}{\underset{i=1}{\sum }}\left(m_{i}\right)G_{x,w}}\right)\\ \;+m_{w}\left(\frac{-\overset{n}{\underset{i=1}{\sum }}\left(\tau_{w,i}m_{i}\right)G_{w,x}^{2}}{{\left(\overset{n}{\underset{i=1}{\sum }}\left(m_{i}\right)+m_{w}G_{w,x}\right)}^{2}}-\frac{\overset{n}{\underset{i=1}{\sum }}\left(\tau_{i,w}m_{i}\right)G_{x,w}}{{\left(m_{w}+\overset{n}{\underset{i=1}{\sum }}\left(m_{i}\right)G_{w,x}\right)}^{2}}\right)\\ \;+\ln\;\left(\frac{1000/Ms}{1000/Ms+\overset{n}{\underset{i=1}{\sum }}\left(m_{i}\right)}\right) \end{array}$$

In this model, Equation (9) is the final objective function. Five parameters (*h*, $\tau_{w,i}^{\left(0\right)}$, $\tau_{w,i}^{\left(1\right)}$, $\tau_{i,w}^{\left(0\right)}$, and $\tau_{i,w}^{\left(1\right)}$) need to be calculated in the equation. Experimental data (Tables 2–6) and the data in the literature (Xu et al., 2014, 2016) were used for correlation. τ_*w,i*_ and τ_*i,w*_ are related to the temperature, and the temperature range is between 298.15 and 440.15 K.

The physical meaning of parameters (*n, m*_*x*_, *m*_*i*_, *m*_*w*_, *h*_*i*_, *n*_*t*_, *Ms*, τ_*w,x*_, τ_*x,w*_, τ_*w,i*_, and τ_*i,w*_) in this model is shown in the NOMENCLATURE. In this model, the reference state of activity coefficients is γ_*i*_ → 1 as *x*_*i*_(=*n*_*i*_*/n*_*t*_) → 1. Five parameters (*h*, $\tau_{w,i}^{\left(0\right)}$, $\tau_{w,i}^{\left(1\right)}$, $\tau_{i,w}^{\left(0\right)}$, and $\tau_{i,w}^{\left(1\right)}$) were fitted to the VLE data for the LiBr + CaCl_2_ + H_2_O system in the final equations. The 1stOpt 7.0 (7D-Soft High Technology Inc.) optimization software was chosen as the main tool for simulation calculations.

## Results and Discussion

**Figure 3:**
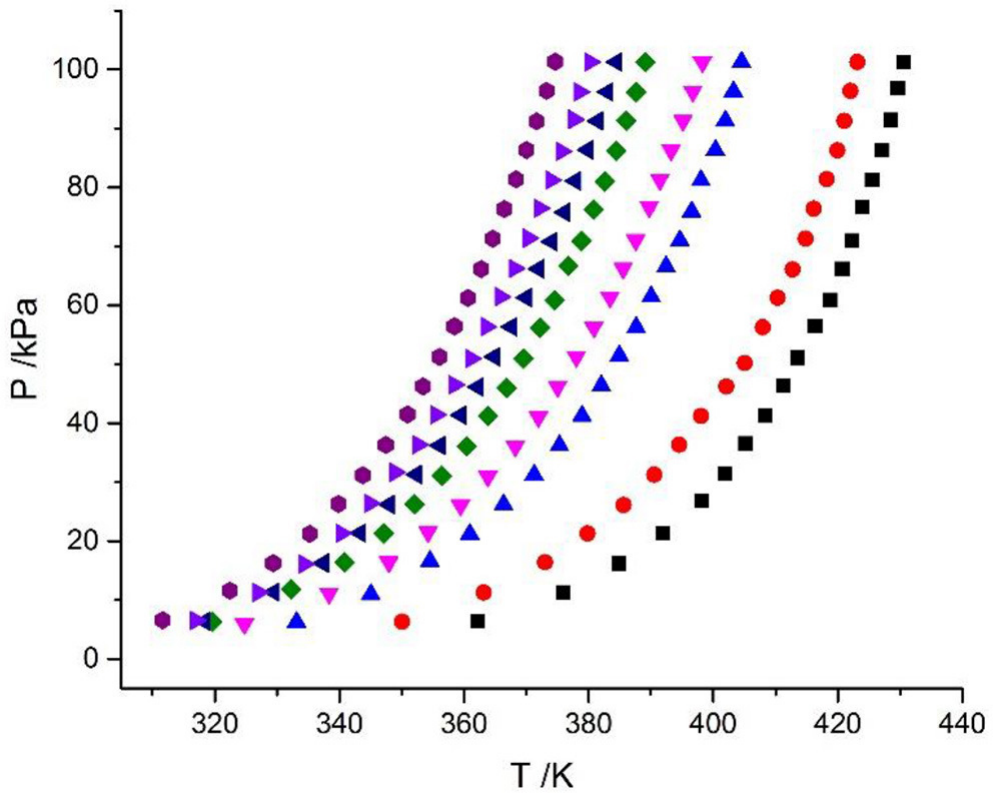
Vapor–liquid equilibrium in the LiBr + H_2_O system. Full symbols (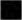, *m* = 21.05 mol/kg; 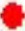, *m* = 16.5 mol/kg; 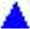, *m* = 11 mol/kg; 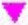, *m* = 9 mol/kg; 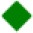, *m* = 7 mol/kg; 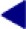, *m* = 5.5 mol/kg; 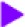, *m* = 4 mol/kg; 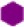, *m* = 3 mol/kg): experimental data.

**Figure 4:**
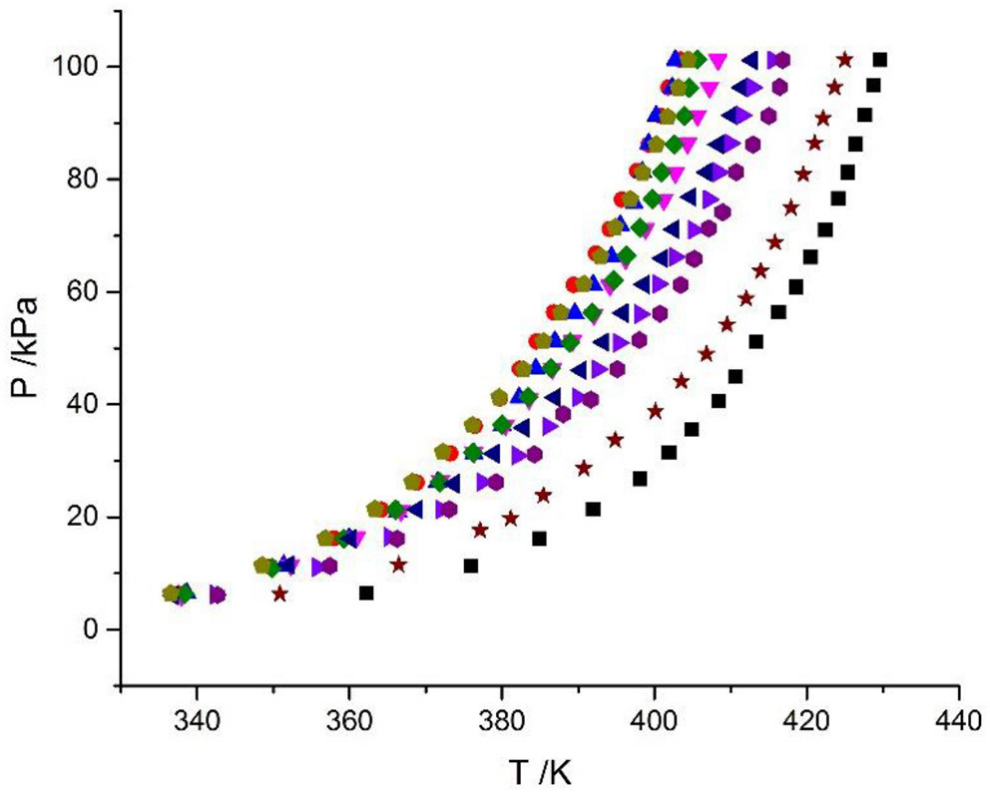
Experimental VLE data for the LiBr + CaCl_2_ + H_2_O system. Symbols (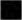, ma = 21.05 mol/kg, mb = 0 mol/kg; 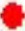, ma = 1.5 mol/kg, mb = 8.5 mol/kg; 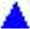, ma = 3.08 mol/kg, mb = 8.1 mol/kg; 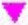, ma = 4.12 mol/kg, mb = 7.1 mol/kg; 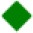, ma = 5.5 mol/kg, mb = 6.1 mol/kg; 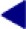, ma = 7.1 mol/kg, mb = 4.7 mol/kg; 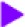, ma = 8.95 mol/kg, mb = 4.08 mol/kg; 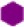, ma = 11 mol/kg, mb = 3.3 mol/kg; 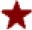, ma = 16.5 mol/kg, mb = 1.5 mol/kg; 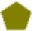, ma = 0 mol/kg, mb = 8.91 mol/kg): experimental data (this work).

In this work, the VLE data of the LiBr + H_2_O and LiBr + CaCl_2_ + H_2_O systems were experimentally measured at concentrations ranging from 0 to 21.05 mol•kg^−1^ and pressures ranging from 6 to 101.3 kPa; the data are listed in Tables 2–6. Analysis and summary of the experimental data are shown in Figures 3, 4.

For the study of the activity coefficient model for electrolyte solutions, we usually choose the activity coefficient of the molality concentration standard. Thus, we only need to study the activity data of water in the electrolyte solutions (Chen et al., 1982; Chen and Evans, 1986; Xu et al., 2014). The Xu model was used to correlate and predict the VLE for the LiBr + H_2_O and LiBr + CaCl_2_ + H_2_O systems. The applicable system of the model was extended in this work. The correlation and prediction results were used to compare the Pitzer model (Pitzer, 1973), ElecNRTL model (Chen et al., 1982; Chen and Evans, 1986), and Xu model, and the VLE behavior of the LiBr + CaCl_2_ + H_2_O system was investigated.

### Discussion of Experimental Results

The LiBr + H_2_O and LiBr + CaCl_2_ + H_2_O systems were chosen to study the VLE, as shown in Tables 2–6 and Figures 3, 4. The tables and figures show that the VLE of LiBr + H_2_O and LiBr + CaCl_2_ + H_2_O are similar. It is well-known that as the salt concentration increases in the LiBr + H_2_O and LiBr + CaCl_2_ + H_2_O systems, the vapor pressure of water decreases. From Tables 2–6 and Figures 3, 4, we can see that the vapor pressure at *m*_LiBr_ = 21.05 mol•kg^−1^ and *m*_CaCl2_ = 0 mol•kg^−1^ in the LiBr + CaCl_2_ + H_2_O system is lowest, and the activity at the corresponding temperature is lowest.

**Figure 5:**
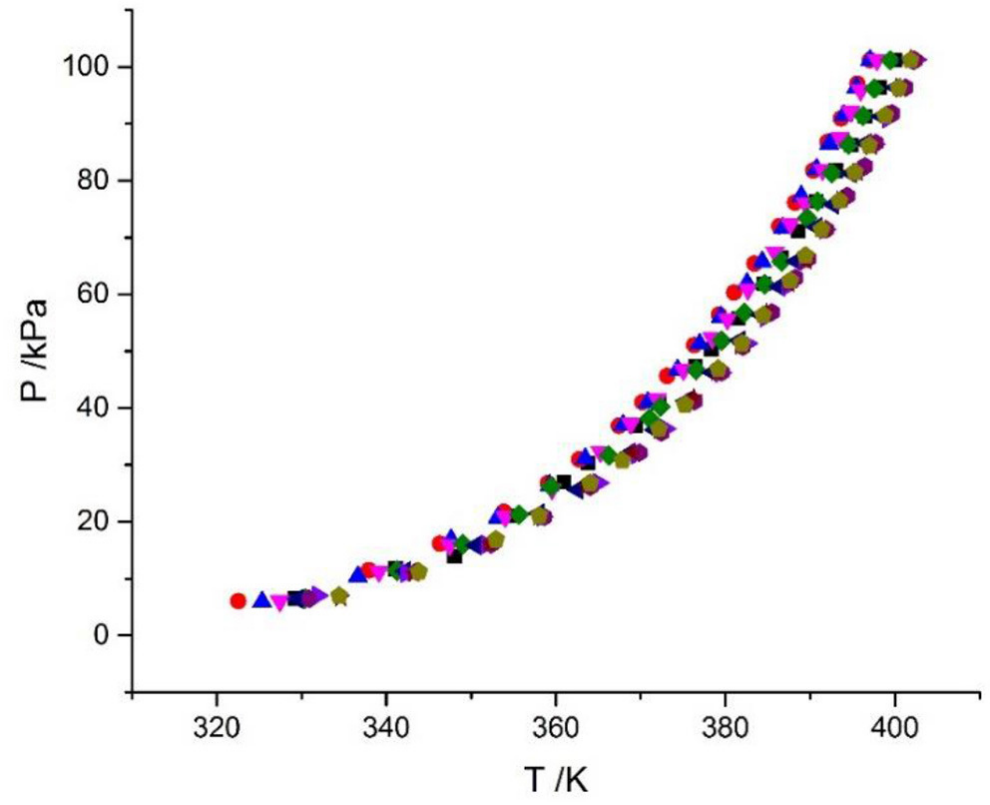
VLE data for the LiCl + CaCl_2_ + H_2_O system (Xu et al., 2019a). Symbols (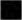, ma = 20.08 mol/kg, mb = 0 mol/kg; 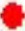, ma = 15.63 mol/kg, mb = 1.3 mol/kg; 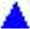, ma = 10.4 mol/kg, mb = 3.14 mol/kg; 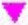, ma = 8.83 mol/kg, mb = 4.07 mol/kg; 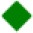, ma = 7.46 mol/kg, mb = 4.68 mol/kg; 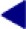, ma = 5.41 mol/kg, mb = 5.95 mol/kg; 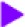, ma = 3.73 mol/kg, mb = 6.73 mol/kg; 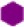, ma = 2.43 mol/kg, mb = 7.08 mol/kg; 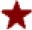, ma = 1.66 mol/kg, mb = 7.14 mol/kg; 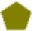, ma = 0 mol/kg, mb = 7.72 mol/kg).

The VLE of the CaCl_2_ + H_2_O, LiBr + H_2_O, LiBr + CaCl_2_ + H_2_O, and LiCl + CaCl_2_ + H_2_O systems are shown in Figures 4, 5. From the figures, it can be known that the LiBr + H_2_O curve at saturated solubility (*m* = 21.05 mol•kg^−1^) and normal temperature is lower than that of CaCl_2_ + H_2_O, LiBr + CaCl_2_ + H_2_O, and LiCl + CaCl_2_ + H_2_O. The LiBr + CaCl_2_ + H_2_O curve at the same concentration and temperature is lower than that of LiCl + CaCl_2_ + H_2_O. Therefore, the hygroscopicity of some systems containing LiBr is also relatively high, and the hygroscopicity of the LiBr + H_2_O system at saturated solubility (*m* = 21.05 mol•kg^−1^) is the highest.

### Results of the Modeling

#### Correlation of the VLE

**Figure 6:**
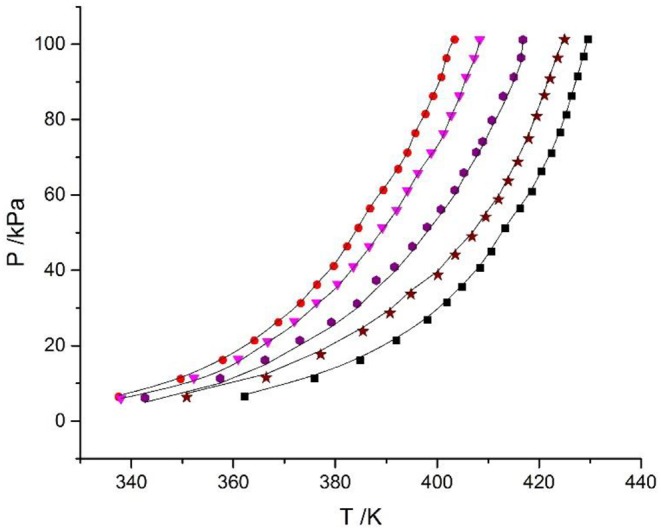
Correlation of experimental VLE data for the LiBr + CaCl_2_ + H_2_O system. Symbols (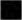, ma = 21.05 mol/kg, mb = 0 mol/kg; 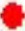, ma = 1.5 mol/kg, mb = 8.5 mol/kg; 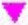, ma = 4.12 mol/kg, mb = 7.1 mol/kg; 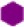, ma = 11 mol/kg, mb = 3.3 mol/kg; 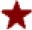, ma = 16.5 mol/kg, mb = 1.5 mol/kg): experimental data (this work); lines: correlation of the model.

Equation (9) was used to correlate VLE data for the LiBr + H_2_O and LiBr + CaCl_2_ + H_2_O systems. The results of the correlation for the LiBr + CaCl_2_ + H_2_O system are shown in Figure 6. The deviation between the literature and the calculated values for the LiBr + H_2_O, CaCl_2_ + H_2_O, and LiBr + CaCl_2_ + H_2_O systems are listed in Table 8. Parameters, $\tau_{1,2}^{0}$, $\tau_{2,1}^{0}$, $\tau_{1,3}^{0}$, $\tau_{3,1}^{0}$, $\tau_{2,3}^{0}$, $\tau_{3,2}^{0}$, $\tau_{1,2}^{1}$, $\tau_{2,1}^{1}$, $\tau_{1,3}^{1}$, $\tau_{3,1}^{1}$, $\tau_{2,3}^{1}$, $\tau_{3,2}^{1}$, *h*_1_, and *h*_2_were obtained from the correlation of the experimental and literature data, as listed in Table 7. For LiBr + CaCl_2_ + H_2_O, it can be seen from Table 8 that *dY* = 0.31 kPa and *dP* = 2.55%. *dY* and *dP* were calculated via the following equations:

(10) $$\begin{array}{l} dY=\left(1/N\right)\sum |P_{\exp}-P_{\text{cal}}| \end{array}$$

(11) $$\begin{array}{l} dP=\left(1/N\right)\sum |P_{\exp}-P_{\text{cal}}|/P_{\exp}\times 100 \end{array}$$

where *N* denotes the number of data points, and *P*_exp_ and *P*_cal_ denote experimental vapor pressure and calculated vapor pressure, respectively.

**Table 7:** Model parameters for the CaCl_2_-H_2_O, LiBr-H_2_O, and LiBr-CaCl_2_-H_2_O systems.

**System**			***a***	***h***	**$\tau_{i,w}^{\left(0\right)}$**	**$\tau_{w,i}^{\left(0\right)}$**	**$\tau_{i,w}^{\left(1\right)}$**	**$\tau_{w,i}^{\left(1\right)}$**
CaCl_2_-H_2_O	CaCl_2_	Reference 4	0.3	1.1	781.44	−3771.77	−98.47	−6010.44
LiBr-H_2_O	LiBr	Correlated in this work	0.3	0.8	−5.47	56.87	510.23	−23,153.41
LiBr-CaCl_2_-H_2_O	LiBr	Correlated in this work	0.3	−25.82	−5.14	−5129.97	−1949.2	2,149,363.27
	CaCl_2_			−66.64	−8.4	4740.2	−1046.8	−1,973,929.6

**Table 8:** Correlation results of VLE data.

**System**	***p* (kPa)**	**Data points**	**This work**		**Data source**
			***dY* (kPa)^a^**	***dP* (%)^b^**	
CaCl_2_-H_2_O	5–101.3	322	0.081	1.82	4, 15
LiBr-H_2_O	5–101.3	180	0.191	2.15	Experiment
LiBr-CaCl_2_-H_2_O	5–101.3	200	0.31	2.55	Experiment

a *dY = (1/N)∑|P_exp_ – P_cal_|, where N is the number of data points*.

b *dP = (1/N)∑|P_exp_ – P_cal_|/P_exp_ × 100%, where N is the number of data points*.

#### Prediction of the VLE

**Figure 7:**
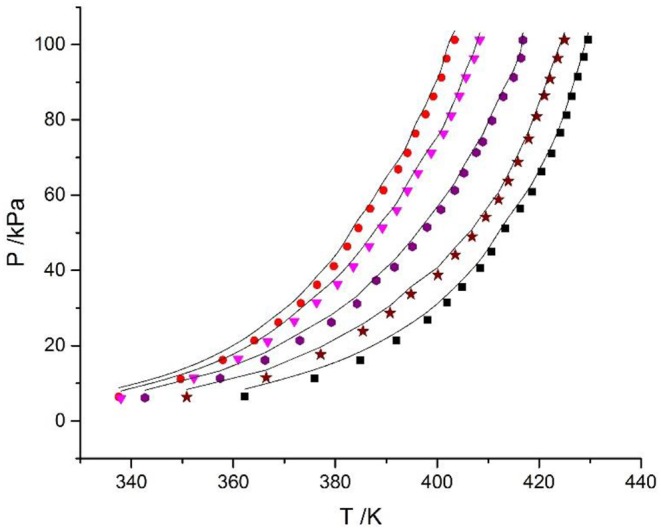
Prediction of experimental VLE data for the LiBr + CaCl_2_ + H_2_O system. Symbols (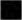, ma = 21.05 mol/kg, mb = 0 mol/kg; 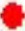, ma = 1.5 mol/kg, mb = 8.5 mol/kg; 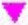, ma = 4.12 mol/kg, mb = 7.1 mol/kg; 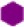, ma = 11 mol/kg, mb = 3.3 mol/kg; 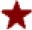, ma = 16.5 mol/kg, mb = 1.5 mol/kg): experimental data (this work); lines: prediction of the model.

The Xu model was chosen to correlate and predict the VLE. In previous work, the model was also successfully applied to predict the VLE data in mixed electrolyte solution systems with binary parameters (Xu et al., 2016). However, the parameters of LiBr are lacking. The prediction parameters of CaCl_2_ were obtained from the literature (Xu et al., 2019a), the parameters of LiBr were calculated using LiBr + H_2_O experimental data in this work, as listed in Table 7. The prediction result is shown in Figure 7, where *dY* = 3.1 kPa and *dP* = 5.96%, which are worse than the correlation results.

### Comparison With Other Methods

For the LiBr + CaCl_2_ + H_2_O system calculation, we choose the ElecNRTL model and Pitzer model for comparison with this work. The Pitzer equation for the thermodynamic properties of electrolytes is developed on the basis of theoretical insights from improved analysis of the Debye-Huckel model. The system of equations developed in the first paper of this series is successfully applied to the available free energy data at room temperature for 227 pure aqueous electrolytes in which one or both ions are univalent. The ElecNRTL model proposed by Chen et al. (1982) is generalized to represent the excess Gibbs energy of aqueous multicomponent electrolyte systems. Using only binary parameters, the model correlates and predicts the deviation from ideality of aqueous multicomponent electrolyte systems over the entire range of temperatures and concentrations. The comparison results are shown in Table 9. Note that the results from both the ElecNRTL and Pitzer models were calculated by the software Aspen Plus 8.1 (Xu et al., 2019a).

**Table 9:** Comparison of models for the electrolyte solutions.

**System**	***p* (kPa)**	**Data points**	**Chen-NRTL**		**Pitzer**		**This work (correlation using the Xu model)**		**This work (prediction using the Xu model)**		**Data source**
			***dY* (kPa)^a^**	***dP* (%)^b^**	***dY* (kPa)^a^**	***dP* (%)^b^**	***dY* (kPa)^a^**	***dP* (%)^b^**	***dY* (kPa)^a^**	***dP* (%)^b^**	
LiBr-CaCl_2_-H_2_O	5–101.3	200	4.1	8.96	2.75	4.51	0.31	2.55	3.1	5.96	Experiment

a *dY = (1/N)∑|P_exp_ – P_cal_|, where N is the number of data points*.

b *dP = (1/N)∑|P_exp_ – P_cal_|/P_exp_ × 100%, where N is the number of data points*.

For the LiBr + CaCl_2_ + H_2_O system, the *dY* value (0.31 kPa) of this work (correlation) using the Xu model is smaller than that of the ElecNRTL model (*dY* = 4.1 kPa) and Pitzer model (*dY* = 2.75 kPa). Likewise, the *dP* value (2.55%) of this work (correlation) is smaller than that of the ElecNRTL model (*dP* = 8.96%) and Pitzer model (*dP* = 4.51%).

In this work, we expand the scope of the model based on previous work (Xu et al., 2014, 2019a). The parameters of the LiBr + H_2_O system were obtained in this paper. Then, the binary parameters of LiBr + H_2_O and CaCl_2_ + H_2_O were used to predict the VLE for the LiBr + CaCl_2_ + H_2_O system. However, the results are not satisfactory. Therefore, we recommend using the correlated parameters of LiBr + CaCl_2_ + H_2_O in Table 8 to calculate the VLE.

## Conclusions

In this paper, VLE data for LiBr + H_2_O and LiBr + CaCl_2_ + H_2_O systems were measured and reported. By the analysis, it is shown that the type and concentration of salt are important factors affecting the VLE. The VLE curve of the LiBr + H_2_O system at saturated solubility (*m* = 21.05 mol•kg^−1^) and 25°C is lower than that of CaCl_2_ + H_2_O, LiBr + CaCl_2_ + H_2_O, and LiCl + CaCl_2_ + H_2_O. The hygroscopicity of some systems containing LiBr is also relatively high, and the hygroscopicity of the LiBr + H_2_O system at saturated solubility (*m* = 21.05 mol•kg^−1^) is the highest.

By correlation of the experimental data, the parameters of the LiBr + H_2_O and LiBr + CaCl_2_ + H_2_O systems were obtained in this paper. The correlation results and prediction results were compared to those of the ElecNRTL and Pitzer model. By comparison, the correlation results of the LiBr + CaCl_2_ + H_2_O system in this work are better than those of the ElecNRTL and Pitzer models. The model can be used to successfully calculate VLE data for LiBr + H_2_O and LiBr + CaCl_2_ + H_2_O systems.

## Data Availability Statement

All datasets generated for this study are included in the article/supplementary material.

## Author Contributions

HW: experimental design and data processing. HC: experimental design and experimental equipment assembly. WC: experimental operation and data processing. HS: data processing and modeling. XX: overall planning of the article and modeling.

### Conflict of Interest

The authors declare that the research was conducted in the absence of any commercial or financial relationships that could be construed as a potential conflict of interest.

## Figures and Tables

**Table:** 

*a*	activity
*G^e^*	excess Gibbs energy, J•mol^–1^
*R*	gas constant, J•mol^–1^•kg^–1^
*m*	molality, mol•kg^–1^
*n*	mole, mol
*m_x_*	total molality of solute, mol•kg^–1^
*m_w_*	molar of free water, mol•kg^–1^
*h*	hydration numbers of the solute
Z	solvation parameters
*T*	temperature, K
*Ms*	molecular weight of water
γ	activity coefficients
*n_t_*	integral molar quantity, mol
τ	parameter
*i*	component *i*
*j*	component *j*
*dY*	mean absolute error, mol•kg^–1^
*dP*	mean relative error, %

## References

[B1] Abrams D. S. Prausnitz J. M. (1975). Statistical thermodynamics of liquid mixtures: a new expression for the excess Gibbs energy of partly or completely miscible systems. AIChE J. 21, 116–128. 10.1002/aic.690210115

[B2] Chen C. C. Britt H. I. Boston J. Evans L. (1982). Local composition model for excess Gibbs energy of electrolyte systems. Part I: Single solvent, single completely dissociated electrolyte systems. AIChE J. 28, 588–596. 10.1002/aic.690280410

[B3] Chen C. C. Evans L. B. (1986). A local composition model for the excess Gibbs energy of aqueous electrolyte systems. AIChE J. 32, 444–454. 10.1002/aic.690320311

[B4] Chua H. Toh H. Malek A. Ng K. Srinivasan K. (2000). Improved thermodynamic property fields of LiBr–H_2_O solution. Int. J. Refrig. 23, 412–429. 10.1016/S0140-7007(99)00076-6

[B5] Filippov V. Mikhelson K. (1977). A thermodynamic study of the system LiCl-CaCl_2_-H_2_O at 25 and 35 C. Zh. Neorg. Khim. 22, 1689–1694.

[B6] Lan Z. Ma X. Hao Z. Jiang R. (2017). Experiments on saturated vapor pressure of aqueous lithium bromide solution at high temperatures. Int. J. Refrig. 76, 73–83. 10.1016/j.ijrefrig.2016.11.025

[B7] Li D. Li S. Meng L. Deng T. Guo Y. (2017). Solid–liquid phase equilibria of ternary systems LiCl–LiBr–H_2_O and CaCl_2_-CaBr_2_-H_2_O at 288.15 K. J. Chem. Eng. Data 62, 833–838. 10.1021/acs.jced.6b00855

[B8] Li D. Zeng D. Han H. Guo L. Yin X. Yao Y. (2015). Phase diagrams and thermochemical modeling of salt lake brine systems. I. LiCl + H_2_O system. Calphad 51, 1–12. 10.1016/j.calphad.2015.05.001

[B9] Li D. Zeng D. Yin X. Han H. Guo L. Yao Y. (2016). Phase diagrams and thermochemical modeling of salt lake brine systems. II. NaCl + H_2_O, KCl + H_2_O, MgCl_2_ + H_2_O and CaCl_2_ + H_2_O systems. Calphad 53, 78–89. 10.1016/j.calphad.2016.03.007

[B10] Lu X. Zhang L. Wang Y. Shi J. Maurer G. (1996). Prediction of activity coefficients of electrolytes in aqueous solutions at high temperatures. Ind. Eng. Chem. Res. 35, 1777–1784. 10.1021/ie950474k

[B11] Lu X. H. Maurer G. (1993). Model for describing activity coefficients in mixed electrolyte aqueous solutions. AIChE J. 39, 1527–1538. 10.1002/aic.690390912

[B12] N'Tsoukpoe K. Le Pierrès N. Luo L. (2013). Experimentation of a LiBr–H_2_O absorption process for long-term solar thermal storage: prototype design and first results. Energy 53, 179–198. 10.1016/j.energy.2013.02.023

[B13] Patek J. Klomfar J. (2006). A computationally effective formulation of the thermodynamic properties of LiBr–H_2_O solutions from 273 to 500 K over full composition range. Int. J. Refrig. 29, 566–578. 10.1016/j.ijrefrig.2005.10.007

[B14] Pitzer K. S. (1973). Thermodynamics of electrolytes. I. Theoretical basis and general equations. J. Phys. Chem. 77, 268–277. 10.1021/j100621a026

[B15] Renon H. Prausnitz J. M. (1968). Local compositions in thermodynamic excess functions for liquid mixtures. AIChE J. 14, 135–144. 10.1002/aic.690140124

[B16] Thomsen K. Rasmussen P. Gani R. (1998). Simulation and optimization of fractional crystallization processes. Chem. Eng. Sci. 53, 1551–1564. 10.1016/S0009-2509(97)00447-8

[B17] Wilson G. M. (1964). Vapor-liquid equilibrium. XI. A new expression for the excess free energy of mixing. J. Am. Chem. Soc. 86, 127–130. 10.1021/ja01056a002

[B18] Xu X. Cao D. Liu J. Gao J. Wang X. (2019c). Research on ultrasound-assisted demulsification/dehydration for crude oil. Ultrason. Sonochem. 57, 185–192. 10.1016/j.ultsonch.2019.05.024 31208613

[B19] Xu X. Gu X. Wang Z. Shatner W. Wang Z. (2019b). Progress, challenges and solutions of research on photosynthetic carbon sequestration efficiency of microalgae. Renew. Sustain. Energy Rev. 110, 65–82. 10.1016/j.rser.2019.04.050

[B20] Xu X. Hu Y. Wang X. Wu L. (2016). Experimental and modeling of vapor–liquid equilibria for mixed electrolyte solution systems. J. Chem. Eng. Data 61, 2311–2320. 10.1021/acs.jced.5b01028

[B21] Xu X. Hu Y. Wu L. Zhang S. (2014). Experimental and modeling of vapor–liquid equilibria for electrolyte solution systems. J. Chem. Eng. Data 59, 3741–3748. 10.1021/je500623w

[B22] Xu X. Wang Y. Sun X. Zhou Y. (2019a). Vapor–liquid equilibria study of the LiCl + CaCl_2_ + H_2_O System. ACS Omega 4, 4390–4396. 10.1021/acsomega.8b03570 31459638 PMC6648044

[B23] Zeng D. Xu W. Voigt W. Xia Y. (2008). Thermodynamic study of the system (LiCl + CaCl + HO). J. Chem. Thermodynamics 40, 1157–1165. 10.1016/j.jct.2008.02.010

